# Strain Measurement in Aluminium Alloy during the Solidification Process Using Embedded Fibre Bragg Gratings

**DOI:** 10.3390/s16111853

**Published:** 2016-11-04

**Authors:** Klaus Weraneck, Florian Heilmeier, Markus Lindner, Moritz Graf, Martin Jakobi, Wolfram Volk, Johannes Roths, Alexander W. Koch

**Affiliations:** 1 Institute for Measurement Systems and Sensor Technology, Technical University of Munich, 80333 Munich, Germany; m.graf@tum.de (M.G.); m.jakobi@tum.de (M.J.); a.w.koch@tum.de (A.W.K.); 2 Chair of Metal Forming and Casting, Technical University of Munich, 85748 Garching, Germany; florian.heilmeier@tum.de (F.H.); wolfram.volk@utg.de (W.V.); 3 Photonics Laboratory, Munich University of Applied Sciences, 80335 Munich, Germany; markus.lindner@hm.edu (M.L.); roths@hm.edu (J.R.)

**Keywords:** fibre bragg grating, aluminium alloy, solidification process

## Abstract

In recent years, the observation of the behaviour of components during the production process and over their life cycle is of increasing importance. Structural health monitoring, for example of carbon composites, is state-of-the-art research. The usage of Fibre Bragg Gratings (FBGs) in this field is of major advantage. Another possible area of application is in foundries. The internal state of melts during the solidification process is of particular interest. By using embedded FBGs, temperature and stress can be monitored during the process. In this work, FBGs were embedded in aluminium alloys in order to observe the occurring strain. Two different FBG positions were chosen in the mould in order to compare its dependence. It was shown that FBGs can withstand the solidification process, although a compression in the range of one percent was measured, which is in agreement with the literature value. Furthermore, different lengths of the gratings were applied, and it was shown that shorter gratings result in more accurate measurements. The obtained results prove that FBGs are applicable as sensors for temperatures up to 740 °C.

## 1. Introduction

Residual stresses in castings arise primarily because of temperature differences and its changes. These are internal loads that are present without the appearance of external forces and torques and that are internally balanced [1]. An aluminium engine block with integrated cylinder liners made of grey cast iron is an example of a compound casting that contains a large amount of residual stresses because of the different thermal expansion coefficients. The resulting disturbances have a huge influence on the behaviour of the component and its life cycle [2]. Knowledge of the condition of residual stresses and their occurrence is of great interest for predicting such disturbances with the help of simulation tools.

Most testing methods are destructive and change the internal balance of the casting because they rely on material removal, which results in the deformation of the component. This can be measured with strain gauges and converted back to residual stresses. However, only the strain on the surface will be recorded, and an analysis of the internal stresses is generally impossible [3].

Non-destructive methods, such as X-ray or neutron diffraction, can determine distance changes in the lattice planes [4]. Besides macroscopic examinations, the behaviour of micro residual stresses has also been studied [5,6]. Numerous studies focus on the analysis of residual stresses caused by various production processes, such as casting [7], welding [8,9] or surface treatment, e.g., deep rolling [10]. The occurring residual stresses during these processes have been further analysed using simulation tools [11,12]. However, the accuracy of their predictions is insufficient, especially for temperature-dependent techniques, such as casting, owing to a lack of experimental data for validation [7,13,14]. The above-mentioned non-destructive methods are expensive and cumbersome. This makes them inappropriate for acquiring these missing experimental data. A suitable measuring method should not affect the behaviour of the material, resist temperatures up to 800 ${}^{\circ }$C and offer the possibility of observing the component non-destructively.

The listed requirements provide the motivation to search for alternative sensing systems. Fibre Bragg Gratings (FBGs) are increasingly the subject of current research and scientific discussions. Their usage offers benefits that allow their integration in modern materials, especially during the production process. The diameter of the fibres is around 125 μm, and so, they can be embedded, for example, in castings with minimal influence on the carrier material. Moreover, the sensors are insensitive to electromagnetic influences and chemically resistant. Recent research topics deal with the application of FBGs in fields, such as aerospace, composite materials, structural health monitoring or high temperature measurements.

An FBG is generated by a refractive index modulation in an optical fibre with a period length Λ of about 500 nm. Owing to this variation of the refractive index *n*, a part of the guided light is reflected in a narrow band around the so-called Bragg wavelength $\lambda_{b}$. The Bragg wavelength is defined by the effective refractive index $n_{eff}$ of the fibre and the grating period Λ: $\lambda_{b}=2n_{eff}\Lambda$ [15,16]. The FBG is sensitive to strain and temperature changes in the fibre, which results in a shift of the Bragg wavelength. This shift can be measured, and consequently, FBGs can be used as strain and temperature sensors [17]. Standard sensors are called Type I FBGs and are generated by ultraviolet (UV) exposure. Their photosensitivity is based on the creation of colour centres and density changes [18]. An overview of the actually-used grating types can be found in [19].

The aim of this work was to embed FBGs during the casting process in a preliminary investigated moulding process to observe the behaviour of the sensors during the solidification process. Different positions and grating lengths were tested and compared. With the aid of additionally placed thermocouples, the effect of temperature on the FBGs was calculated, and conclusions about the strain appearing that occurs during the solidification process can be drawn. The acquisition of temperature data up to 800 ${}^{\circ }$C in melts with temperature-stable FBGs has already been performed [20], but those fibre sensors were not used to measure strain in order to analyse the residual stresses.

In this work, it was shown that Type I FBGs can withstand high temperatures without an inordinate amount of degradation of the gratings, and conclusions about the strain can be drawn.

## 2. Measurement Principle of FBGs

**Figure 1:**
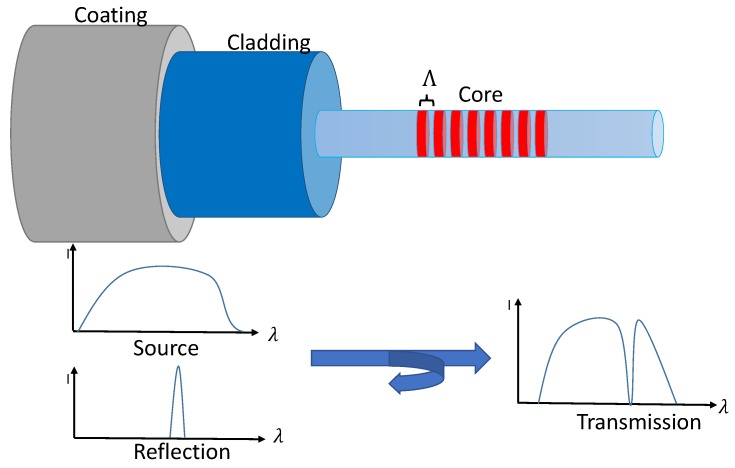
Structure of a Fibre Bragg Grating and its measurement principle.

FBGs are a type of spectral coded sensor, as their evaluation can be performed via intensity analysis, as well as resonance wavelength analysis. This resonance wavelength is called Bragg wavelength $\lambda_{b}$ and is sensitive to changes in temperature and elongation Δl. In Figure 1, the structure of an optical fibre with an FBG is illustrated. The core of the fibre contains a periodic modulation of the refractive index [21]. If a broadband light source is used as the input, part of the light will be reflected, and the rest is transmitted. If there is an elongation of the part that contains the index modulation, e.g., caused by a strain *ϵ*, the grating period Λ undergoes a linear geometric change that results in a shift of the reflection. In addition, the refractive index changes, which can be described by the opto-mechanic effect. This effect enables the measurement of the applied longitudinal strain *ϵ*.

Another possible effect that can be measured by this sensor is the change in temperature. This causes an alteration of the grating period through thermal expansion of the fibre and also modifies the refractive index. Both effects initiate a shift of the Bragg wavelength and can be correlated to the change in temperature. A linear approach of the shift of the Bragg wavelength in a free fibre due to the mentioned temperature effect and strain along the fibre axis can be seen in Equation (1) [17].

(1) $$d\lambda =\lambda_{b}\left[\frac{1}{n_{eff}}\frac{\delta n_{eff}}{\delta \epsilon }+\frac{1}{\Lambda }\frac{\delta \Lambda }{\delta \epsilon }\right]d\epsilon +\lambda_{b}\left[\frac{1}{n_{eff}}\frac{\delta n_{eff}}{\delta T}+\frac{1}{\Lambda }\frac{\delta \Lambda }{\delta T}\right]dT$$

The first part of the equation handles the impact of the strain and the second part the temperature effect. When a grating is exposed to strain along the fibre in the z-axis, the relation between the shift in wavelength Δλ and the strains $\epsilon_{x}$, $\epsilon_{y}$ and $\epsilon_{z}$ can be described by Equation (2) [22].

(2) $$\frac{\Delta \lambda_{b}}{\lambda_{b}}=\epsilon_{z}-\frac{n_{eff}^{2}}{2}\left[p_{11}\epsilon_{x}+p_{12}\left(\epsilon_{y}+\epsilon_{z}\right)\right]$$

For the next step, axis-symmetric homogeneous strain is assumed, where the radial strain $\epsilon_{x}$ is the same as $\epsilon_{y}$, and the lateral strains are related to axial strain by a standard Poisson number: $\epsilon_{x}=\epsilon_{y}=-\nu \epsilon_{z}$. In the case of a small variation of the fibre length, the differential dis assumed to be the difference Δ. Based on the linear relation between the fibre length and the modification of the grating period, the term δΛ/Λ passes over to δϵ. Equation (2) simplifies to [23,24]:

(3) $$\frac{\Delta \lambda_{b}}{\lambda_{b}}=\left(1-P\right)\Delta \epsilon$$

where P = $\frac{1}{2}n_{eff}^{2}\left(p_{12}-\nu \left(p_{11}+p_{12}\right)\right)$ and is called the photoelastic coefficient. This coefficient depends on Pockel’s coefficients $p_{11}$ and $p_{12}$ and on the Poisson number *ν*. Regarding the values of a characteristic optical fibre, the values for Pranges from 0.205–0.230. These assumptions can be made because the connection between the fibre and the aluminium shows imperfections, and due to this, the influence of the strain in the axial direction will be outweighed. The cross-sections of the probes can be seen later in this work, and encourage this simplification. Nevertheless this aspect has to be considered when a method for a perfect fibre-aluminium connection is found.

Assuming small temperature variations, its influence can be described with Equation (4).

(4) $$\frac{\Delta \lambda_{b}}{\lambda_{b}}=\left(\alpha +\gamma \right)\Delta T$$

The thermal expansion coefficient *α* can be specified as $\alpha =0.55\times 10^{-6}$ K${}^{-1}$ [23] for Ge-doped silica glass, and the thermo-optic constant *γ* is around $8.3\times 10^{-6}$ K${}^{-1}$ [25]. As the second value is one magnitude higher, the impact of the refractive index change caused by temperature variations is the dominant effect. For temperatures higher than 700 ${}^{\circ }$C in an aluminium melt, the above-stated simplification cannot be used.

**Figure 2:**
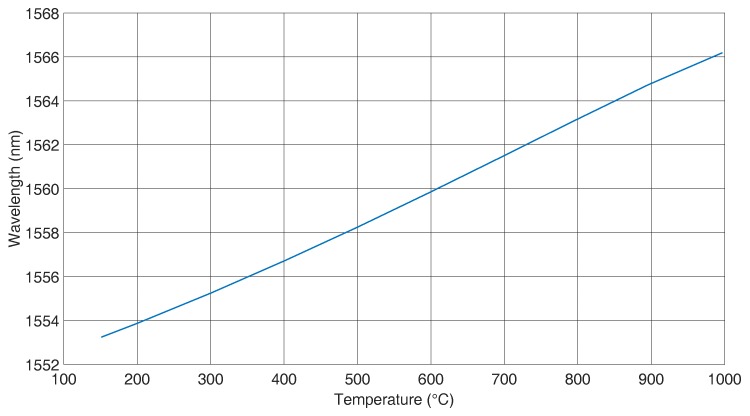
Temperature calibration curve of a regenerated FBG in a GF1B fibre.

In this work, photosensitive fibres of Type GF1B (Nufern) were used, and Type I FBGs with reflectivities in the order of 80%–90% were inscribed by employing the phase mask technique and a 248 nm laser source. The used fibres were not $H_{2}$ loaded. One Type I FBG was regenerated [26], and with this grating temperature, calibration measurements between room temperature and 1000 ${}^{\circ }$C were performed using a high temperature oven “Pegasus plus 1200” from Isotech GmbH, Germany. During temperature calibration, the FBG was free floating inside one pocket of the oven, and the fibre was free of any external mechanical loads. Figure 2 shows the temperature calibration data.

A cubic polynomial was fitted to the data, which is given by the following Equation. This polynomial fitting results in more precise data than a linear fitting [27]. In addition, the temperature dependence of the expansion coefficient is also included in this calculation.

(5) $$\lambda \left(T\right)=A+B*T+C*T^{2}+D*T^{3}$$

The fit parameters are compiled in Table 1, when *T* is given in ${}^{\circ }$C.

For the casting experiments, Type I FBGs in GF1B fibre without regeneration were used because they showed a better mechanical stability [29]. For this reason, no other type of FBG was used for these tests. In this study of temperature and strain over time curves, we made the assumption that the temperature calibration curve of the regenerated FBG can also be applied to Type I FBGs that were inscribed into the same type of optical fibre. It is known that this will cause an error, but the measurement results verify that this error is in an acceptable range. At high temperatures, degradation and wavelength drifts were observed in the Type I FBGs. However, during the casting process the fibre is only for several seconds at temperatures above 600 ${}^{\circ }$C and only for around 20 min at temperatures in the range between 200 ${}^{\circ }$C and 600 ${}^{\circ }$C. It was found that for this limited temperature load, Type I FBGs can be employed, but for the considerations of the measurement uncertainty, temperature-induced wavelength drifts have to be taken into account. For wavelength drift and degradation evaluation, we performed reference measurements with capsuled Type I FBGs that were installed during similar casting processes as those described in Section 3. After the casting process, all of test samples of encapsulated FBGs still had more than 20% reflectivity, which was sufficient for Bragg wavelength determination. The Bragg wavelengths of the encapsulated FBGs had been measured at room temperature before the casting process and again after the casting process when the cast part was cooled down to room temperature again. The wavelength drift was found to be less than 150 pm in all samples, which corresponds to circa 15 ${}^{\circ }$C in the temperature reading. This wavelength drift is assumed to be the dominant part of the measurement uncertainty, but this is acceptable because it is still small relative to the observed wavelength changes of the embedded FBGs, as can be seen in Section 4. The strain sensitivity of FBGs in GF1B optical fibre at room temperature was reported in [26,30] to be:

(6) $$\frac{\Delta \lambda }{\lambda }=k=0.789$$

In this study, we assume that the strain sensitivity is independent of temperature.

**Figure 3:**
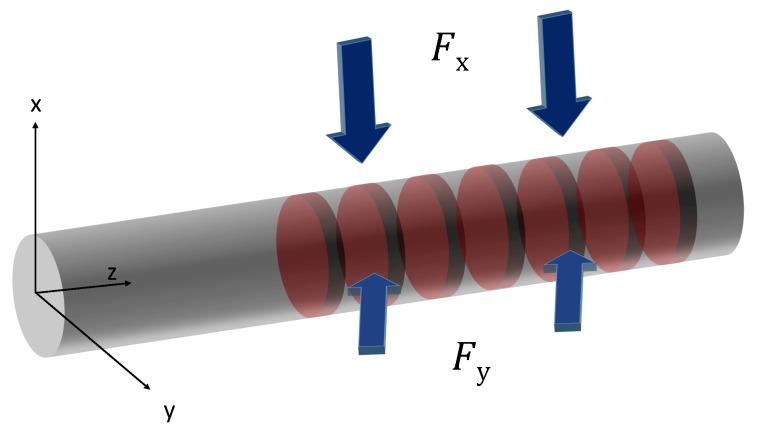
Two different strains on an optical fibre cause birefringence.

FBGs are sensitive to temperature and strain, so that a compensation method is needed if just one of these parameters is required. For an embedded FBG, the internal strain matters, and therefore, a method to measure the temperature has to be found. For example, a second test point can be equipped with a thermocouple, and its data are used to calculate the influence of the temperature. It is important that the reference point be as near as possible to the original test point or be placed in a reference probe under the same conditions. After this is done, the recorded strain can be determined. Another characteristic has to be explained when embedding FBGs into materials: amorphous solid materials, such as glass or plastics, are generally isotropic. Hence, only one refractive index $n_{0}$ appears in an optical fibre. In Figure 3, two different transversal strains $\epsilon_{x}$ and $\epsilon_{y}$ are present. For an FBG in a single-mode fibre, these varying strains induce two local dependent refractive indexes [31,32]. This leads to a separation of the reflection from one into two peaks and is called birefringence [33,34].

With knowledge of the strains in the corresponding axes, the two shifts can be calculated using Equations (7) and (8) [35,36].

(7) $$\frac{\Delta \lambda_{b,x}}{\lambda_{b}}=\epsilon_{z}-\frac{n_{0}^{2}}{2}\left[p_{11}\epsilon_{x}+p_{12}\left(\epsilon_{y}-\epsilon_{z}\right)\right]$$

(8) $$\frac{\Delta \lambda_{b,y}}{\lambda_{b}}=\epsilon_{z}-\frac{n_{0}^{2}}{2}\left[p_{11}\epsilon_{y}+p_{12}\left(\epsilon_{x}-\epsilon_{z}\right)\right]$$

The separation in this case is proportional to the difference between the transversal strains $\epsilon_{x}$ and $\epsilon_{y}$, and the mathematical correlation is given by Equation (9).

(9) $$\frac{\Delta \lambda_{b,y}-\Delta \lambda_{b,x}}{\lambda_{b}}=\frac{n_{0}^{2}}{2}\left(p_{12}-p_{11}\right)\left(\epsilon_{y}-\epsilon_{x}\right)$$

## 3. Material and Methods

**Figure 4:**
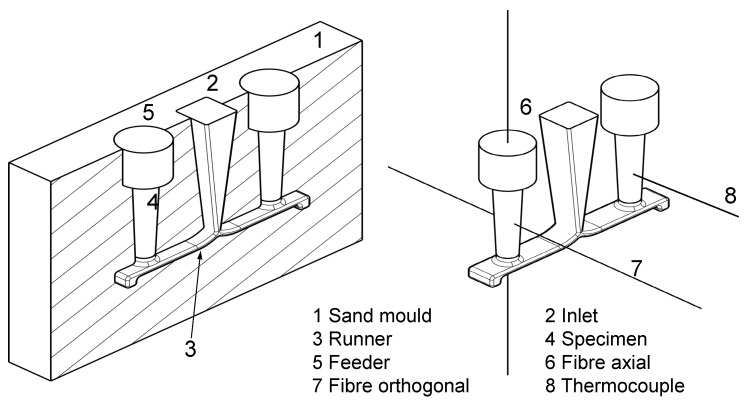
Components of the casting mould.

For casting processes, a mould that keeps the liquid alloy in place is needed. For the experiments, a hand-formed sand mould was used, as shown in Figure 4. Two mould halves form the cavity, which consists of a runner and a feeding system for casting aluminium specimens. The mould allows one to place the fibre in axial and orthogonal direction and, thus, measure in two independent directions. For each measurement, only one FBG was used to avoid the disturbance impact on the probe being too large. Another reason for the solution with one sensor was to avoid any mutual interactions during the solidification process. The fibres were fixed in the mould with the help of stainless steel capillaries. The outside diameters of the capillaries were 0.8 mm. In each case, two of them were used, and the FBG was located between these elements. As the difference between the ends of the capillaries and the FBG is more than 15 times the capillary diameter, there is no influence of the capillaries on the measurement [37]. There are several advantages of this fixing method. Firstly, the capillaries are robust and can be plugged into the hardened sand without destroying either the form or the capillaries. Secondly, they enable the fibres to be fixed without the risk of breaking them. Thirdly, they protect the fibre during the casting process. With every mould, there are two specimens, as can be seen in Figure 4. With the aid of the second symmetrical specimen, a reference measurement of the temperature can be conducted. The symmetrical form allows us to assume that heat distribution behaviour will be uniform. The reference measurements were performed with a k-type thermocouple. The form filling process lasted about two seconds at 740 ${}^{\circ }$C. The composition of the standardized aluminium alloy is given in Table 2 (DIN EN 1706:2010).

**Figure 5:**
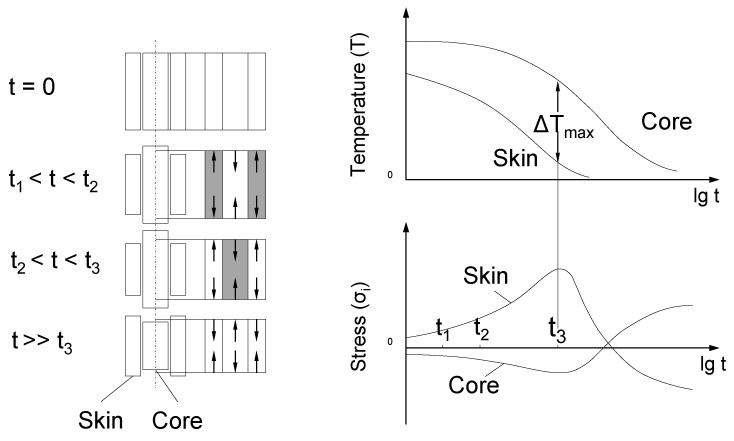
Tension conversion and formation of residual stresses [1].

In the left panel of Figure 4, one sand mould (1) can be seen. The alloy is distributed through the inlet (2) and the runner (3) to the specimen (4). The feeder (5) serves as a buffer for the specimen. During solidification, a shrinkage of around 1% can be observed. To avoid defective spots, such as cavities in the specimen, the shrinkage is compensated by the feeder. On the right side of Figure 4, the two different positions of the fibre can be seen. The reference thermocouple is placed in the symmetrical specimen. The FBG is located in the middle of the specimen and has a length between three and four mm. The fibre position marked with 6 is oriented along the melt flow direction, and that marked with 7 is oriented across the melt flow direction. To understand the solidification procedure in the probe, some points regarding residual stresses should be stated. In this work, the thermal residual stresses are the most important. During a quick cooling and solidification process of a metallic object, there is a temperature difference between the core and the skin, thus causing Type I residual stresses [1]. The probe in Figure 5 is assumed to be a thermally and elastically isotropic, homogeneous and conversion-free cylindrical object. The upper right graph in Figure 5 illustrates ΔT over time. The left part of Figure 5 indicates that the initially more contracting skin part causes tensile stress at the borders and compressive stress in the areas near the core. The lower right graph in Figure 5 shows the stress curves of the core and the skin. If ΔT reaches a specific value, the tensions override the yield point of the material, and plastic deformation starts at the borders.

It is restricted to the borders if the height of the cylindrical body is larger than its diameter (l/d>4), because the disability of the flow in the core will occur due to the multi-axis system. After reaching the maximum temperature difference at time $t_{3}$, the plastic deformation stops for the moment. The beginning heat adjustment minimizes tensions at the border and compressions in the core areas until conversion of the mechanical tensions starts. This does not appear simultaneously in the whole cross-section. After this conversion, especially the core shrinks. Thus, there are simultaneous compressive stresses at the border and tensile stresses near the core after a balance of the temperature is achieved. This behaviour is typical for thermal residual stresses.

**Figure 6:**
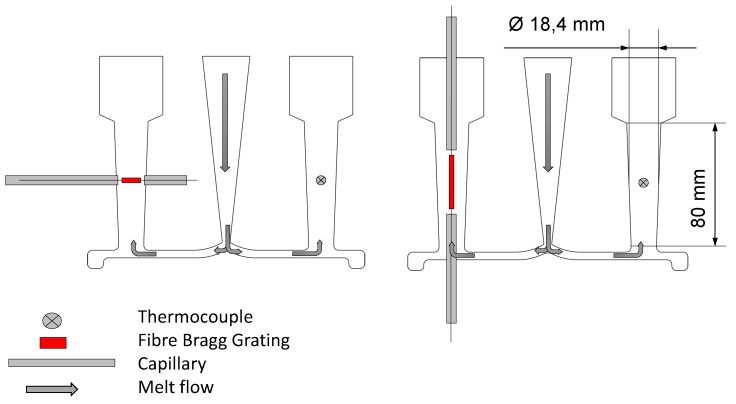
The two different positions of the FBGs in the probes.

At the end of this section, the influence of the FBG position in the melt is examined in detail. Figure 6 shows both the melt flow and the two different locations of FBGs. The dimensions of the probe are also given.

In the left panel of Figure 6, the FBG is fixed across the material flow, and in contrast to this position, it is located along the flow in the right part. Both methods are extended with a thermocouple in the reference path for the compensation of the temperature influence on the fibre. The ends of the FBGs are protected by capillaries for reasons already stated. The set-up in the right panel was chosen, because it was supposed that in this situation, a homogeneous strain field around the sensor would be created. The other position was chosen based on two facts: the first reason is that during the inlet of the melt, there is a great force on the fibre, and it should be proven that the fibre can withstand this; the second aspect is that this rectangular fixing offers a good comparison to the fibre placed along it.

## 4. Results and Discussion

Three different aspects of the solidification and measurement process are considered and discussed here.

### 4.1. Measurements during the Solidification Process

**Figure 7:**
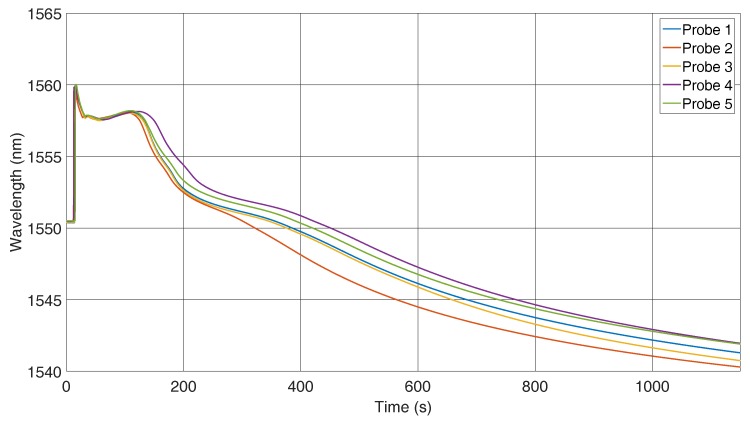
Peak wavelength of the reflected spectra of the FBGs across the flow direction.

**Figure 8:**
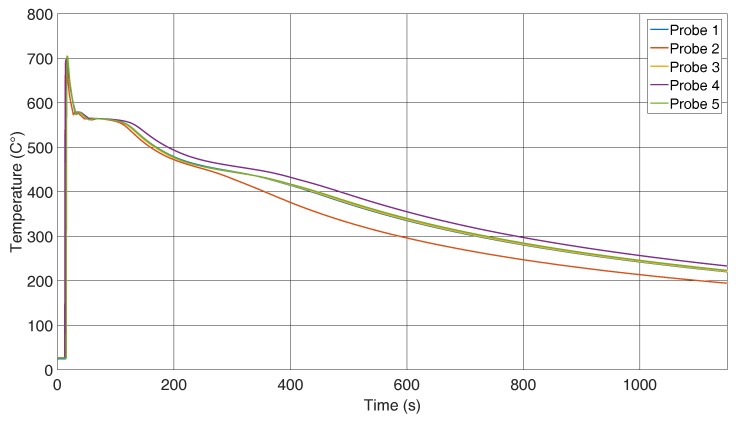
Temperature in the reference specimen.

**Figure 9:**
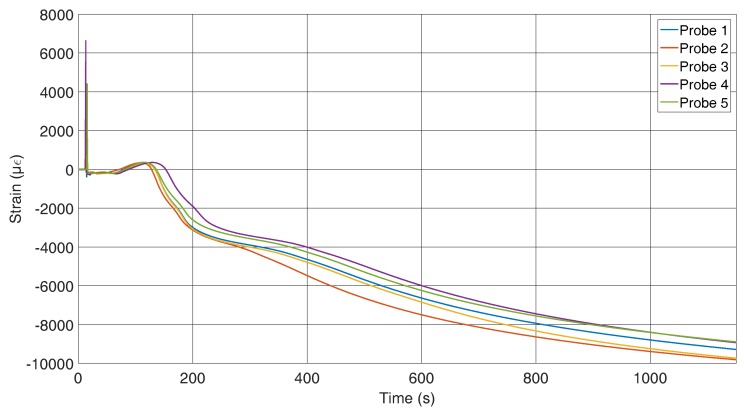
Strain curves during the solidification process measured by FBGs across the flow direction of the melt.

In this section, the results of the position-dependent measurements are shown. Figure 7, Figure 8 and Figure 9 refer to experiments where the FBG was located across the material flow direction, as shown in Figure 6. Five castings were executed with this FBG position, and all of the FBGs used had the same specifications. A measuring time of 1150 s was chosen because the temperature in the reference path reaches 200 ${}^{\circ }$C within this span. At this point, it is certain that the solidification process will have finished. Initially, the reflected spectra show the same peak, which changes noticeably after 120 s, where we have a clear differentiation, that can be seen in Figure 7, which shows the wavelength shift caused by the strain and temperature changes.

To find the reason behind this phenomenon, each temperature has to be taken into account. Figure 8 illustrates the temperature in the reference path, and as stated previously, it can be assumed that the same temperature is present around the fibre. The casting temperature was around 740 ${}^{\circ }$C, and at the first contact with the thermocouple, it had already cooled to 700 ${}^{\circ }$C. During the cooling process down to 200 ${}^{\circ }$C, a difference that is caused by different effects can be seen. The greatest discrepancy was caused by fabrication of the sand moulds. They are made manually, and hence, it cannot be guaranteed that the same force was always applied when compressing the sand. This difference causes variations in the thermal conductivity and establishes a gap between the five probes. A second impact is the different environmental conditions. Drafts and the outside ambient temperatures could not be controlled during the test. The third known reason is the filling quantity, which differs in every casting. The more melt inserted into a form, the higher the heat capacity of the specimen. This causes a reduced cooling rate for probes with a higher amount of melt.

The curve with 1 in Figure 8 corresponds to the 1 in Figure 7. The comparison of all curves indicates that the mismatch between peak wavelengths is caused by the various cooling rates of the probes. As explained before, if the temperature is known, the applied strain on the sensor can be calculated. In Figure 9, peak shift and temperature were used to calculate the strain. The measured strain represents an average over the grating length, but due to the small grating length, its behaviour can be considered as a point measurement.

The outlier values within the first few seconds can be explained by the first contact with the aluminium alloy. The alloy possesses a surface tension that stretches the fibre to a specific point at which the fibre breaks through. Another source of error is the discrepancy between the response time of the FBG and the thermocouple. In our set-up, the FBG response is faster or is in contact with the alloy earlier, thus inducing a shift of the wavelength owing to an increase of temperature. The thermocouple reacts later to the alloy or the time delay is due to the differences in position. Since the data from the thermocouple are used for the FBG compensation, this delay may result in the peak that is seen in the graph. The unit of strain is chosen as μϵ and is equal to μϵ = μm/m. After the first contact with the aluminium, the sensor measures a compression that can be explained by an oxide layer that docks on the fibre. Additionally breaking the surface tension of the melt could also influence the strain measurement. Subsequently, the strain increases until a maximum is reached at a time of 1600 s. This corresponds to the tension conversion, as can be seen in Figure 5. Thereafter, the impact of the shrinkage of the aluminium predominates, and the strain converts into compression. After 1150 s, a strain of almost −10,000 μϵ is reached, which is equal to a shrinkage of about one percent. This is in agreement with the specifications of the alloy [39].

**Figure 10:**
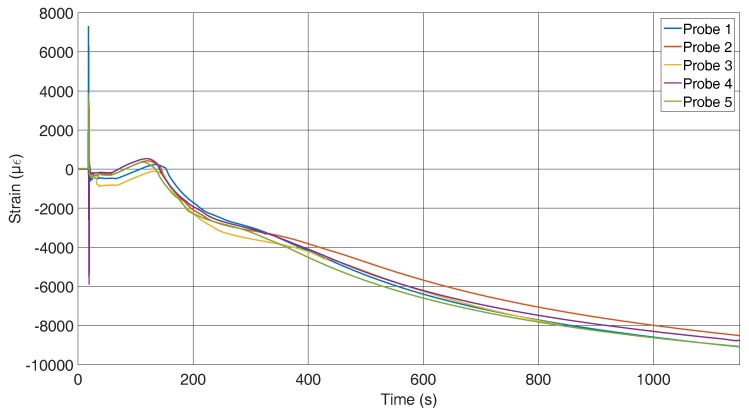
Strain curves during the solidification process measured by FBGs along the flow direction of the melt.

Figure 10 shows the results of the strain measurements of the specimens where the fibre is mounted in the flow direction of the melt. The initial outliers have the same origin as mentioned for the other sensor position. The data up to the tension conversion show a larger deviation than those for the across-fixed fibres. To explain this, the cross-sections of the specimens have to be compared, which is presented later in the paper. The strains after the strain conversion are comparable to those measured with the first set-up.

**Figure 11:**
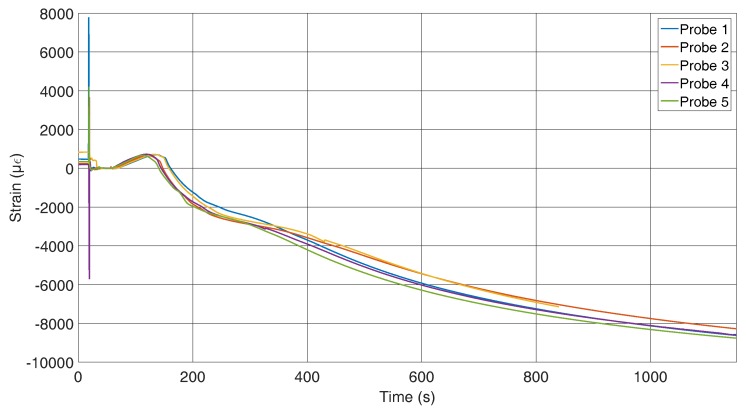
Strain along the fibre with offset.

In comparison, the fixation across the flow direction shows less deviation between the first contact with the melt and the tension conversion. The first contact with the melt induces an additional change in the wavelength for the probes with the fibres along the flow due to the fixation. For those probes, a pre-stress is applied to the sensors to achieve a straight position; thus, it seems that this pre-stress disappears because of the inlet. To obtain a comparable graph to the strain graph for the across located sensors in Figure 11, the strain for all probes of Figure 10 were set to zero after the inlet. It can now be seen that the probes behave very similarly and that the assumption that the temperature influences the pre-stress is correct.

### 4.2. Comparison of the Fibre-Alloy Connection

**Figure 12:**
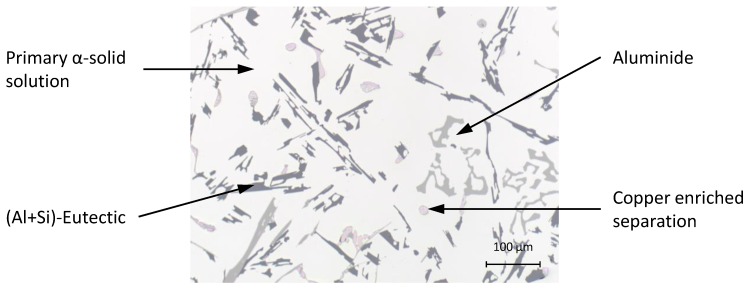
Cross-section of an aluminium alloy probe without a fibre.

Comparison of the sensor fixations revealed a difference in the strain values. To find the reason for this behaviour, the cross-sections of the different probes had to be examined. In Figure 12, a cross-section of a probe without a fibre can be seen.

**Figure 13:**
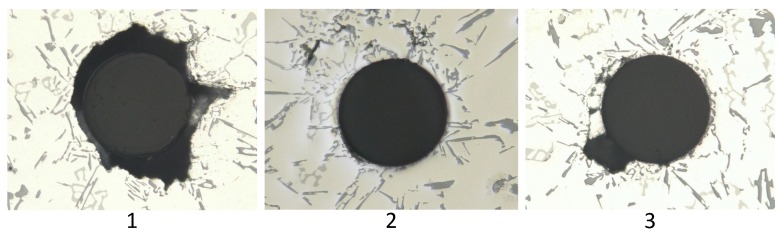
Cross-sections of three different probes where the fibre is located along the melt flow direction.

**Figure 14:**
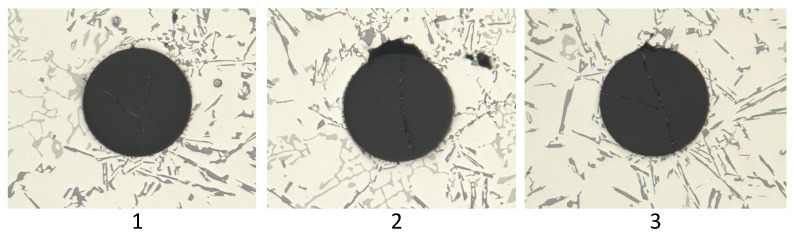
Cross-sections of three different probes where the fibre is located across the melt flow direction.

Figure 13 shows three cross-sections of probes with fibres along the flow direction. Figure 14 illustrates the cross-sections of across-fixed fibres. The black areas around the fibres, especially in the first panel of Figure 13, present imperfections, such as gas bubbles or vacuum cavities. In addition, these imperfections cause the formation of an oxide layer around the fibre, which cannot be seen in the three parts of Figure 13 because of its small dimensions. This additional layer has its own transfer function, and thus, the strain transfer can differ. Another disruptive factor also has to be analysed because the oxide layer is not homogeneously attached around the fibre, which can initiate birefringence. As the shown cross-sections present only a small part of the probe and the measurement data still indicate strain, it is certain that these imperfections do not cover the whole FBG.

In the case of the position across the flow, the interface quality between the fibre and the aluminium alloy was much better. The connection is depending on the orientation regarding the material flow direction. The part of the fibre that is directly met by the melt offers a better connection because gas bubbles did not attach to it. In contrast, the imperfections tended to adhere to the other side of the fibre. Hence, the strain field was homogeneous at the bottom of the fibre and inhomogeneous on the other side. These inhomogeneities are restricted to a small area and, thus, did not disturb the measurement significantly.

For the position along the flow, there is no preferred direction of the defects apparent. They tended to appear around the upper part of the sensor, but no statistical statement about their exact position can be made; as explained before, the oxide layer disturbs the force transfer to the fibre and falsifies the strain. There are two different reasons for these imperfections. The insertion of the melt induces the generation of turbulences in the fluid and, thereby, gas bubbles. During the cooling of the melt, gases such as hydrogen separate from the metal and rise. These gas bubbles can ascend in the feeder or the specimens and are random.

Comparison of the cross-sections of the probes proves that both fixing methods produce an interface with the solidified alloy that guarantees a strain transfer to the sensor. However, the quality of the strain transfer is affected by the sensor positioning, which means that the fibre across the flow direction generated less impurities and, hence, an almost homogeneous strain field around the sensor. This has two advantages. Firstly, the transfer function is not distorted through the additional oxide layer. Secondly, if the strain around the fibre is not anisotropic, no birefringence will occur, and so, only the strain along the fibre is detected.

### 4.3. Impact of the Grating Length

**Figure 15:**
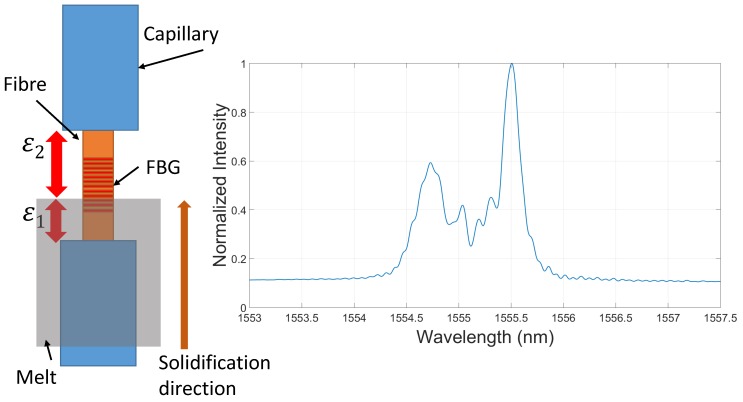
Deformation of the reflected spectra due to the influence of different strains along the fibre.

Besides the mentioned tests with a grating length of around three to four mm, additional set-ups with a grating length of 8 mm and a position along the melt flow were done. For the further consideration, it is assumed that the melt solidifies along the fibre. In the left panel of Figure 15, solidification along the fibre is presented in a simplified way. The solidification front arises from the bottom up to the top and, because of the grating length of eight mm, the sensor divides into two different parts. Now, the strains $\epsilon_{1}$ and $\epsilon_{2}$ form the reflected spectrum of the FBG that can be seen in the right part of Figure 15. This multi-strain behaviour can be observed also after the solidification process for 8-mm gratings. To understand the problem, the algorithm to analyse the data has to be taken into account. The reflection is fitted with a Kaiser window followed by a peak-finding, which finds the maximum value. Thereby, only one value is used to calculate the strain. That means only one strain is observed, which does not correspond to the complete strain along the fibre, and besides that, the two strains interact, which creates an extra error.

**Figure 16:**
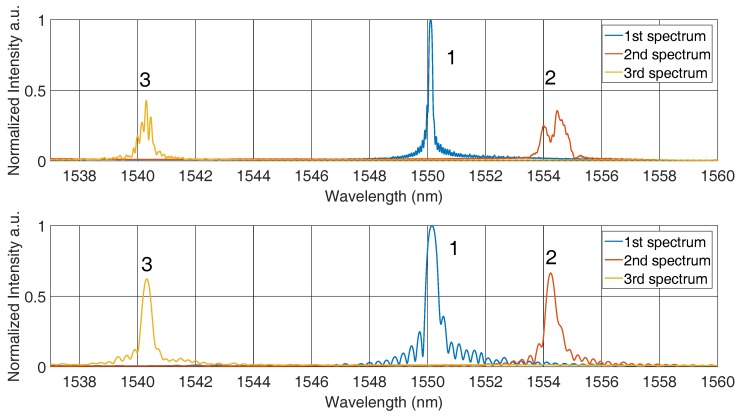
Comparison of the reflected spectra of an 8-mm FBG (upper graph) and a shorter FBG with a length of 3–4 mm.

It can be expected that the real situation is even more complicated, and so, there can be more strain fields along the fibre. This increases with a gain of the grating length. To prove this theory, the spectra of the 3–4 mm and 8 mm spectra were compared at the same moments with respect to this. Figure 16 shows the changes in the reflected spectra during the solidification and cooling process. The first spectrum was recorded before the casting. For this situation, the 8 mm FBG in the upper graph shows one peak, as can be seen for the 3–4 mm sensor in the lower graph. The width of the spectrum for the 3 mm sensor is greater because of the less gratings. The second spectrum is taken a while after the melt was inserted and at a temperature of around 500 ${}^{\circ }$C. In the upper panel of Figure 16, the spectrum is divided, which corresponds to the expected error due to multi-strain measurement. For the shorter sensor, a loss of intensity can be recognized, but still one peak can be clearly detected. The loss in intensity is caused by the decay of the grating at high temperatures. The third spectrum at a temperature of around 200 ${}^{\circ }$C is located at a lower wavelength than the first one because of their compression through the shrinkage of the carrier material.

As expected, the spectra differ significantly at the end of the test cycle and at a temperature of around 200 ${}^{\circ }$C. For a sensor length of 8 mm, more peaks can be seen. Depending on the analysing algorithm used on the spectrum, the Bragg wavelength will differ significantly. If this effect of peak splitting is not taken into account, the conclusion regarding the strain will be inaccurate. Comparison of the two sensor lengths proves that shorter gratings should be preferred and a length of 3–4 mm is a suitable solution.

## 5. Conclusions

The presented experiments and results show the applicability of FBGs in foundries. Installation across the flow direction, as well as along the flow direction provided comparable results. In both cases, a shrinkage of around one percent was measured, which is in agreement with theoretical values. The internal temperature during the cooling process was indicated to be a process parameter with a great deviation of over 50 ${}^{\circ }$C. The influence of the different cooling rates has to be compared in the next steps of this ongoing project.

The cross-sections of the specimens demonstrate that the connection between the fibre and the aluminium alloy is not free from contaminations. This prevents homogeneous force transfer to the sensor. We assume that these weaknesses are the reason for a distortion of the FBG reflection shape. Besides these impurities around the fibre, it can happen that rising gas bubbles surround the FBG, impeding the detection of strain because there is no direct interaction between the sensor and the carrier. A possible solution for these problems is a metallic coating around the fibre. It is anticipated that a copper or aluminium coating should reject almost all impurities, and thus, a homogeneous force field around the fibre shows up. In this case, birefringence may be avoided. The set-up with the sensor position across the melt flow minimizes the formation of an oxide layer and restricts it to one side of the fibre. In addition, rising gas bubbles did not attach themselves to the fibre in this position. Furthermore, the first contact with the melt removed the applied pre-stress of the along placed fibres that causes a shift in wavelength. However, it was shown that this could be regulated by setting an offset that sets all strains to the same value after the first contact. Based on these advantages, this set-up will be used for further measurements.

The data up to 200 ${}^{\circ }$C provided sufficient intensities of the reflection, and the degradation of the gratings is in an acceptable range, so that the peak-finding algorithm works properly. For conclusions with respect to the use of the sensors up to room temperature and beyond this point, long-time experiments have to be executed. One plausible scenario is the degradation of the grating; thus, no further structural health monitoring could be performed. This would require alternative FBGs that can withstand high temperatures over a long time. Regenerated FBGs offer a new approach for this problem, because due to special fibres and production methods, they degenerate in a minimal range. For the long-time experiments, calibration tests for Type I FBGs will be done before. Thus, the error caused by using another calibration curve will be eliminated.

Concerning the length of the sensor, it can be pointed out that shorter FBGs are preferred because of their ability to monitor a local internal state. The longer they get, the higher is the risk that the spectrum splits and more than one peak will appear. In addition to this, the influence of the fibre length, which is exposed to the melt, has to be compared. For an observation of more than one measurement spot in the specimen, different gratings can be inscribed, and so, a distributed strain analysis is realized. In general, the measurement results prove that FBGs offer an alternative to the often-used neutron diffractometry because the acquired data imply the same behaviour for all probes.

## Figures and Tables

**Table 1:** Specifications of the GF1B fibre [28].

A (nm)	B (nm/°C)	C (nm/°C^2^)	D (nm/°C^3^)
1551.669	0.00894	1.131 × $10^{-5}$	−5.63 × $10^{-9}$

**Table 2:** Composition of the aluminium alloy [38].

	%Cr	%Mn	%Zn	%Si	%Cu	%Ni	%Fe	%Mg	%Pb	%Sn	%Ti
AlSi9Cu3	0.15	0.55	1.20	8.00–11.00	2.00–4.00	0.55	1.30	0.05–0.55	0.35	0.25	0.25

## References

[1] Macherauch E., Wolfahrt H., Wolfstieg U. (1973). Zur zweckmäßigen Definition von Eigenspannungen. HTM.

[2] Dye D., Stone H., Reed R. (2001). Intergranular and interphase microstresses. Curr. Opin. Solid State Mater. Sci..

[3] Keil S. (1998). Beanspruchungsermittlung mit Dehnungsmeßstreifen. Bautechnik.

[4] Hutchings M.T., Withers P.J., Holden T.M., Lorentzen T. (2007). Introduction to the Characerization of Residual Stress by Neutron Diffraction. Mater. Characteriz..

[5] Repper J.N. (2010). Einfluss Mikroskopischer Eigenspannungen auf Die Makroskopische Eigenspannungsanalyse Mittels Neutronenbeugung. Ph.D. Thesis.

[6] Withers P., Bhadeshia H. (2001). Residual stress. Part 1–Measurement techniques. Mater. Sci. Technol..

[7] Wasmut U. (2009). Orts- und zeitabhängige Analyse von Eigenspannungen in Verbundguss. Ph.D. Thesis.

[8] Mraz L., Karlsson L., Mikula P., Vrána M. (2013). Identification of Weld Residual Stresses Using Diffraction Methods and their Effect on Fatigue Strength of High Strength Steels Welds. Mater. Sci. Forum.

[9] Ohms C. (2013). Residual Stresses in Thick Bi-metallic Fusion Welds: A Neutron Diffraction Study. Ph.D. Thesis.

[10] Altenberger I., Nikitin I., Juijerm P., Scholtes B. (2006). Residual Stress Stability in High Temperature Fatigued Mechanically Surface Treated Metallic Materials. Mater. Sci. Forum.

[11] Egner-Walter A. (1998). Simulation des Entstehens von Eigenspannungen in Gussteilen.

[12] Hofer P., Kaschnitz E., Schumacher P. (2012). Messung und Simulation von Verzug und Eigenspannungen in Druckgussteilen. Gießerei.

[13] Fent A. (2002). Einfluss der Wärmebehandlung auf den Eigenspannungszustand von Aluminiumgussteilen. Ph.D. Thesis.

[14] Wasmuth U., Meier L., Hofmann M., Mühlbauer M., Stege V., Hoffmann H. (2008). Optimisation of composite castings by means of neutron measurements. CIRP Ann. Manuf. Technol..

[15] Kashyap R. (2009). Fibre Bragg Gratings.

[16] Zeh T., Meixner A., Koch A.W., Neumann C. (2003). Fibre Optic Measurement System for Spatially Distributed Strain and Temperature Measurements. Technisch. Mess..

[17] Kersey A.D., Davis M.A., Patrick H.J., LeBlanc M., Koo K.P. (1997). Fibre Grating Sensors. J. Lightwave Technol..

[18] Limberger H.G., Fonjallaz P.Y., Salathè R.P., Cochet F. (1996). Compaction- and photoelastic-induced index changes in fibre Bragg gratings. Appl. Phys. Lett..

[19] Canning J. (2008). Fibre gratings and devices for sensors and lasers. Laser Photon. Rev..

[20] Heiberg G., Skaar J., Fokine M., Arnberg L. (2002). A new method for temperature measurement in solidifying alloys by use of optical fibre Bragg grating sensors. Trans. Am. Foundry Soc..

[21] Rößner M.R., Müller M.S., Buck T.C., Koch A.W. (2012). Broadband light source for fibre-optic measurement system in spaceborne applications. Acta Astronaut..

[22] Lai M., Karalekas D., Botsis J. (2013). On the Effects of the Lateral Strains on the Fibre Bragg Grating Response. Sensors.

[23] Lindner E. (2012). Erzeugung und Eigenschaften Hoch-Temperaturstabiler Faser-Bragg-Gitter. Ph.D. Thesis.

[24] Mueller U.C., Baier H., Zeh T., Mueller M.S., Koch A.W. (2010). Vibration and Shape Control in Opto-mechanical Systems Using Distributed Fibre-optic Bragg Grating Sensors. J. Vib. Control.

[25] Morey W.W., Meltz G., Glenn W.H., DePaula R.P., Udd E. (1990). Fibre Optic Bragg Grating Sensors. Fibre Optic and Laser Sensors VII.

[26] Polz L., Nguyen Q., Bartelt H., Roths J. (2014). Fibre Bragg gratings in hydrogen-loaded photosensitive fibre with two regeneration regimes. Opt. Commun..

[27] Pal S., Sun T., Grattan K.T., Wade S.A., Collins S.F., Baxter G.W., Dussardier B., Monnom G. (2004). Non-linear temperature dependence of Bragg gratings written in different fibres, optimised for sensor applications over a wide range of temperatures. Sens. Actuators A Phys..

[28] Nufern. http://www.nufern.com/pam/optical_fibres/901/GF1B.

[29] Wang T., Shao L.Y., Canning J., Cook K. (2013). Temperature and strain characterization of regenerated gratings. Opt. Lett..

[30] Jülich F., Aulbach L., Wilfert A., Kratzer P., Kuttler R., Roths J. (2013). Gauge factors of fibre Bragg grating strain sensors in different types of optical fibres. Meas. Sci. Technol..

[31] Othonos A., Kalli K. (1999). Fibre Bragg Gratings: Fundamentals and Applications in Telecommunications and Sensing.

[32] Werneck M., Allil R., Ribeiro B., de Nazare F. (2013). A Guide to Fibre Bragg Grating Sensors. Current Trends in Short- and Long-Period Fibre Gratings.

[33] Wang Y., Yun B., Chen N., Cui Y. (2006). Characterization of a high birefringence fibre Bragg grating sensor subjected to non-homogeneous transverse strain fields. Meas. Sci. Technol..

[34] Müller M.S., Buck T.C., El-Khozondar H.J., Koch A.W. Measurement errors from internal shear strain within fibre-Bragg-grating sensors. Proceedings of the SPIE 7390, Modeling Aspects in Optical Metrology II.

[35] Urban F., Kadlec J., Vlach R., Kuchta R. (2010). Design of a Pressure Sensor Based on Optical Fibre Bragg Grating Lateral Deformation. Sensors.

[36] Gafsi R., El-Sherif M.A. (2000). Analysis of Induced-Birefringence Effects on Fibre Bragg Gratings. Opt. Fibre Technol..

[37] Howland R.C.J. (1930). On the Stresses in the Neighbourhood of a Circular Hole in a Strip under Tension. Philos. Trans. R. Soc. A Math. Phys. Eng. Sci..

[38] AuerGuss Spezifikationen der Aluminium-Legierungen. http://www.auer-guss.de/fileadmin/user_upload/PDF/Legierungstabelle.pdf.

[39] Otremba M. (2015). Standardisierungsaspekte bei der Gießtechnologieauswahl von Zylinderköpfen. Ph.D. Thesis.

